# Preparation and functional evaluation of monoclonal antibodies targeting Hepatitis B Virus Polymerase

**DOI:** 10.1080/21505594.2020.1869391

**Published:** 2021-01-18

**Authors:** Song Hu, Hualong Xiong, Xiaozhen Kang, Shaojuan Wang, Tianying Zhang, Quan Yuan, Deying Tian

**Affiliations:** a Tongji Hospital, Tongji Medical College, Huazhong University of Science and Technology, Wuhan, Hubei Province, China; b School of Life Science & School of Public Health; State Key Laboratory of Molecular Vaccinology and Molecular Diagnostics; National Institute of Diagnostics and Vaccine Development in Infectious Diseases, Xiamen University, Xiamen, China

**Keywords:** HBV, polymerase, antibody, detection, therapy

## Abstract

HBV pol plays a critical role in the replication of hepatitis B virus (HBV). Previous studies conducted on HBV pol have produced limited evidence on HBV pol expression due to the lack of effective detection methods. The present study used the HBV pol (159–406 aa) protein as a target to screen for specific monoclonal antibodies that recognize HBV pol and subsequently evaluate their diagnostic and therapeutic value. Four antibodies (P3, P5, P12, P20) against HBV pol were obtained. Among them, the P20 antibody indicated optimal binding with HBV pol as demonstrated by Western blotting (WB) in a cell model transfected with the HBV genome. We also expressed P5 and P12 antibodies in mouse liver cells by transfection and the results indicated significant antiviral effects caused by these two antibodies especially P12. In summary, the present study established an antibody which was denoted P20. This antibody can be used to detect HBV pol expression by four HBV genomes via WB analysis. In addition, the antibody denoted P12 could exert antiviral effects via intracellular expression, which may provide a promising approach for the treatment of chronic hepatitis B.

## Introduction

HBV can constantly replicate and infect liver cells and is considered an important pathogenic factor that seriously endangers human health [1,2]. Multiple intracellular viral proteins, such as HBx, HBcAg and HBV polymerase, are critical for HBV replication. HBV pol is one of the HBV intracellular proteins involved in almost the entire HBV viral replication process [3,4]. This enzyme is essential for the life cycle of the virus and regulates several processes including viral RNA binding, RNA packaging, protein initiation, template conversion, DNA synthesis and RNA degradation [5]. Nucleos(t)ide analogs (NAs) can act on the polymerase domain and inhibit its activity, resulting in decreased HBV DNA levels [6]. In addition, several recent studies have reported that HBV polymerase interacts directly with STING and RIG-I, thereby interfering with the replication of viral DNA and RNA through pattern recognition receptors in the host immune system [7–9]. HBV pol ultimately reduces the type I interferon response [10–13]. These investigations may provide potential evidence for a therapeutic approach for HBV. Therefore, it is important to study the role of HBV pol in chronic HBV infection. However, the current research field lacks specific and high-quality antibodies for detecting HBV pol, which may hinder further research. In the present study, the HBV pol (159–406 aa) protein was used as a target for the development of specific monoclonal antibodies that recognize native HBV pol and the antiviral effects of the selected antibodies were evaluated.

## Materials and methods

### Plasmid construction

The B-type HBV1.3 ploidy was used as a template, based on the full-length sequence of the genotype B *HBV pol* gene (GU357842) obtained from Genbank. The primers were designed to amplify the *HBV pol* (159–406) DNA fragment. The sequence of the forward primer with a BamHI restriction site at the 5ʹ end was the following: 5ʹ- TGCACCACCACCACCACCACGGATCCATGAAAAGAGAGTCCACACGTAG −3ʹ and that of the reverse primer with an EcoRI restriction site at the 5ʹ end was as follows: 5ʹ- CAAGCTTGTCGACGGAGCTCGAATTCTTATTTTGGCCAAGACACACGGGTGTTC-3ʹ. A 6-His tag series was added to the beginning of the *HBV pol* (159–406) and was ligated to the pTO-T7 plasmid vector.

### Protein expression and purification

The 6His-HBV pol (159–406 aa) protein was expressed following the induction of IPTG and the formation of the inclusion bodies in *E. coli*. A urea buffer was used containing Tris-HCl (4 M urea and 20 mM Tris-HCl pH 8.0 buffer) in order to wash and dissolve the inclusion bodies. The fusion protein was purified by the Ni + affinity chromatography (GE Healthcare, according to the manufacturer’s protocol) and stored at 1 mg/ml at −20 °C.

### Acquisition of mAbs

MAbs against the 6His-HBV pol (159–406 aa) protein were produced using hybridoma technology. A total of 4 antibodies (P3, P5, P12, P20) were selected for evaluation in our research. A mAb (16G12) specific to the HIV-1 p24 protein was selected as a control. A commercial mAb (2 C8) was used as positive control.

### Construction of recombinant antibodies

To construct a recombinant mAb expression plasmid, recombinant antibodies and 16G12 variable genes were obtained by reverse transcription PCR using a mouse Ig primer set (Merck millipore, Darmstadt, Germany). The heavy and kappa chains of the two mAbs were cloned into a pTT5 vector containing the mouse IgG1 constant region (24). Following transfection of the cells with the recombinant antibody plasmid, the cell supernatant was used to detect the activity of HBV pol by indirect chemiluminescence enzyme immunoassay (CLEIA).

### Cell models

HepG2 and Huh7 are hepatoblastoma cell lines, whereas HepAD38 cells expresses HBV genotype (D genome) and HepG2.2.15 expresses HBV genotype (D genome). These cell lines were cultured in DMEM at 37°C [14]. The medium was supplemented with 10% FBS, 100 IU/ml penicillin and 100 IU/ml streptomycin. The cells were cultured in fresh medium every third day, and split by trypsinization at a confluence of approximately 90%. HepAD38 cells, which are a variant of HepG2 cells, expressed the HBV genome under the control of a tetracycline (Tet)-off promoter. Therefore, the HepAD38 cell culture medium also contained tetracycline (1 μg/ml) when the expression of the HBV genes was not required. HepG2.2.15 cells are secretory HBV cells derived from the HepG2 cell line. Transient transfections with plasmids were performed using Lipofectamine 2000 Reagent (Invitrogen, USA) according to the manufacturer’s protocol. The following plasmids were used: Ptt22-HBV1.3, pAAV-HBV1.2, Ptt22-HBV pol and the control plasmid (Ptt22-mcherry). The preparation of these plasmids has been detailed in a previous study [15]. pTSMP-HBV1.3 plasmids with four different genotypes were a kind gift from yongwu, all of which expressed full length HBV [16].

### Mouse models

The hydrodynamic injection (HDI)-based HBV-carrier models were constructed by hydrodynamic tail vein injection using the pAAV-HBV1.2 plasmid. All mice were maintained under speciﬁc pathogen-free conditions in the Laboratory Animal Center of Xiamen University. The experiments were conducted in accordance with the Guide for the Care and Use of Laboratory Animals.

### Viral and immunological assays

To determine the HBV pol (159–406 aa)-binding activities of mAbs, a chemiluminescence enzyme immunoassay (CLEIA) was used. Briefly, wells were coated with recombinant HBV pol (159–406 aa) at a concentration of 100 ng per well and nonspecific binding was blocked with PBS containing 2% (vol/vol) bovine serum albumin (BSA) and 10% sucrose. A series of 3-fold dilutions were prepared. The first antibody concentration was 10,000 ng/ml and the purified antibody was diluted 3 times by 12 gradients. A total of 100 μl specimens were added to the reaction wells and incubated for 1 h at 37°C, followed by washing and reaction with horseradish peroxidase (HRP)-conjugated anti-mouse pAb (Thermo Scientific, USA). The relative light unit (RLU) was evaluated using an Orion II Microplate Luminometer (Berthold, Germany) following the addition of SuperSignal ELISA Pico Chemiluminescent Substrate (Thermo Scientific, USA). The reactivity of recombinant mAbs with HBV pol (159–406 aa) was also measured by CLEIA. The difference was mainly due to the sample containing the culture supernatant of Huh7 cells transfected with recombinant mAb expression plasmid. Immunofluorescence and Western blot analyses for intracellular antibodies were performed according to standard procedures. Viral antigens (HBsAg, HBeAg and HBcAg) were measured using the chemiluminescence method with commercial assay kits (Wantai, Beijing, China). For detection of HBcAg, supernatants (50 μl) were treated with a suitable lysis buffer (50 μl, 20 mM HEPES, 0.5% (vol/vol) Triton X-114, 100 mM NaCl, pH 8.0) at room temperature for 30 min. Subsequently, the mixture was subjected to immunoassay quantification. HBV DNA quantification assays were performed using a commercial real-time PCR kit (Kehua, Shanghai, China). Southern blot was performed according to the methods described previously using DIG-labeled DNA fragments from the X gene as a probe [17].

### Statistical analysis

The data were analyzed by the GraphPad Prism software (version 6.01). A P value lower than 0.05 (P < 0.05) was considered for significant differences.

### Abbreviations

HBV: hepatitis B virus; HBx: hepatitis B x protein; HBsAg: hepatitis B surface antigen; HBeAg: hepatitis B e antigen; HBcAg: hepatitis B core antigen; HBV pol:hepatitis B viruspolymerase; STING: stimulator of interferon genes; RIG-I: The retinoic acid-inducible gene I;IPTG; isopropyl-β-D-1-thiogalactopyranoside

## Results

**Figure 1.**
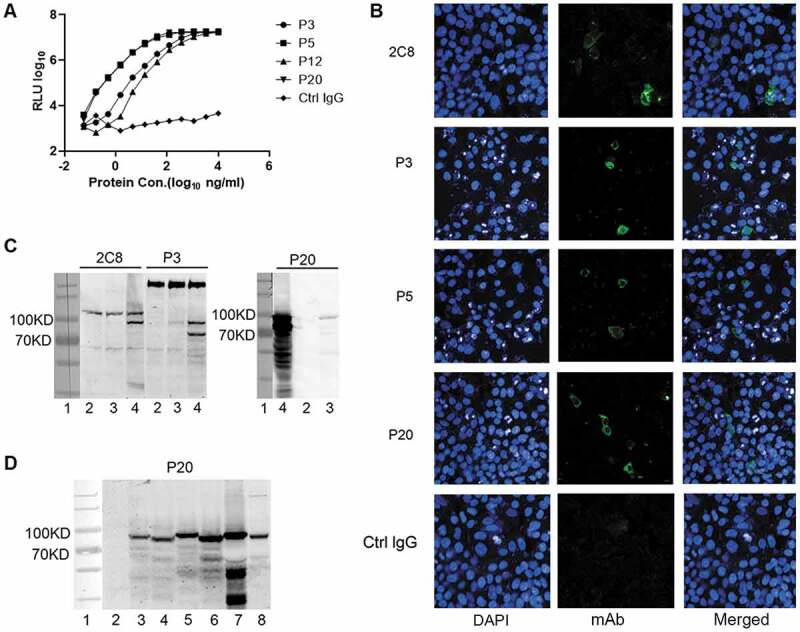
Characterization of the mAb specific for HBV pol (**A**). Kinetic analysis of antibodies against recombinant HBV pol (159–406 aa) using the 3-fold dilution method;(**B**). Immunofluorescence analysis of antibodies against full-length HBV pol in HepG2 cells transfected with HBV pol-expressing plasmids using the mAb specific for HBV pol.;(**C**). WB analysis of antibodies against HBV pol in HepG2 cells transfected with full-length HBV pol-expressing or HBV-expressing plasmids using the mAb specific for HBV pol. Lane 1: Marker; Lane 2: Ptt22-mcherry plasmid (vector); Lane 3: Ptt22-HBV1.3 plasmid; Lane 4: Ptt22-HBV pol plasmid; (**D**). WB analysis of antibody P20 against different genotypes of HBV pol in HepG2 cells transfected with HBV-expressing plasmid or in the HBV cell line. Lane 1: Marker; Lane 2:Ptt22-mcherry plasmid (vector); Lane 3: PTSMP-A plasmid; Lane 4: PTSMP-B plasmid; Lane 5: PTSMP-C plasmid; Lane 6:PTSMP-D plasmid; Lane 7: HepAD38 cells; Lane 8: HepG2.2.15 cells

In the present study, we demonstrated that the selected antibodies (P3, P5, P12, P20) exhibited optimal reactivity with the HBV pol (159–406) recombinant antigen (Figure 1A). However, the recombinant antigen of our immunized mice was a denatured protein with a His tag. Therefore, we further evaluated whether the selected antibodies could react with natural HBV pol. HepG2 cells were transfected with the pTT22-*HBV pol* plasmid, which expressed the full length *HBV pol* gene. Immunofluorescence and WB analysis were used to detect the binding activity of the antibody to the full-length HBV pol. The 2 C8 antibody was used as a control. Immunofluorescence assays demonstrated that the P3, P5 and P20 antibodies indicated binding affinity to the full-length HBV pol expressed by the eukaryotic system (Figure 1B). However, P12 was not reactive (data not shown). Both P3 and P20 antibodies indicated WB reactivity with the full-length HBV pol. Notably P3 exhibited optimal specificity. The P3 antibody exhibited an extra band compared with that of the 2C8 antibody, which may have been caused by the epitope difference between these two antibodies (Figure 1C). The P3 antibody also exhibited a band at 250–300 (estimated) kDa size in all samples tested (Figure 1C). We considered this to be a nonspecific band because the same band was present in all lanes, including the control. The findings may provide a tool for studying the different functions of HBV pol. Although the specificity of the P20 antibody was poor, it exhibited an ideal detection effect on HBV pol, which was expressed in cells transfected with the HBV genome (Figure 1C). Previous studies have not reported an antibody with similar affinity as that in the current study. To confirm our results, we used four genotypes (A, B, C, D) of HBV 1.3 ploidy plasmid-transfected cells and two HBV cell lines (HepG2.2.15 and HepAD38) to verify the detection of the P20 antibody. The results indicated that the detection effect of the P20 antibody was very significant and that it exhibited optimal specificity for the four genotypes of HBV pol (Figure 1D).

**Figure 2.**
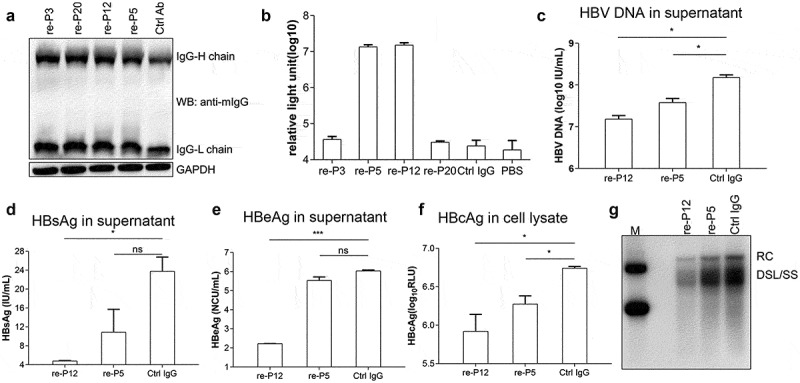
Evaluation of recombinant antibodies expressed in cells by transfection (**A**). Western blot analyses of antibody expressions of Huh7 cells transfected with recombinant mAb expressing plasmids (**B**). The reactivity of recombinant mAbs with HBV pol (159–406 aa) was examined by CLEIA. The sample is the culture supernatant of Huh7 cells transfected with recombinant mAb expression plasmid. PBS: Phosphate buffered saline. (**C**–**G**). Analyses of HBV suppressive effect of plasmid transfection-mediated intracellular expression of recombinant mAbs in HBV-transfected Huh7 cells. The levels of HBsAg, HBeAg and HBVDNA in the supernatants were quantitatively measured by commercial assays. The levels of HBcAg in cell lysates were also quantitatively measured. Southern blot assays were used to assess the effects of mAb treatment on HBV DNA replication in cell lysates. RC: relaxed-circular DNA; DSL: double-strand linear DNA; SS: single-stranded DNA. The data represent mean ± SD. P values were calculated using a two-sided unpaired t test, * indicates P < 0.05, **Indicates P < 0.01, ***Indicates P < 0.001 and ns indicates P > 0.05

To study the antiviral effects of these antibodies, we evaluated their activity in cell and mouse models, respectively. HBV pol is not a secreted protein and it is mainly found in the nucleocapsid of the virus in liver cells. Therefore, we ligated the mAb genes on the pTT22 plasmid vector and subsequently transfected them into the cells in order to express these four antibodies. The antibodies were named re-P3, re-P5, re-P12 and re-P20. Western blot analysis of antibody expression in Huh7 cells transfected with recombinant mAb expression plasmid showed that the recombinant antibodies expressed successfully IgG (Figure 2A).The reactivity of these antibodies was tested with HBV pol (159–406) and the results indicated that re-P5 and re-P12 could specifically react with HBV pol (159–406) compared with the control antibodies. re-P3 and re-P20 were not reactive, which may be caused by different modifications of these antibodies occurring in different cellular environments (Figure 2B).

In order to confirm whether the expression of anti-HBV pol antibodies in the cells exhibited an anti-HBV effect, we co-transfected pTT22-P5 and pTT22-P12 plasmids with the pAAV-HBV1.2 plasmid into Huh7 cells separately. After 3 days, the supernatant of the culture medium was obtained to detect the virology index. The results indicated that P12 could significantly reduce HBsAg, HBeAg, and HBV DNA in the culture medium (Figure 2C and E). It also exhibited an inhibitory effect on HBcAg in the intracellular region (Figure 2F). Viral DNA from cell lysates was isolated and subjected to Southern blot analysis. The levels of replicative HBV DNA intermediates (including RC/DSL DNA and SS DNA) in cell lysates transfected with recombinant anti-HBV pol antibodies were reduced to Ctr-Ab (Figure 2G). In contrast to these findings, re-P5 could not reduce HBsAg and HBeAg levels in the culture medium. The results indicated that anti-HBV pol antibodies could inhibit HBV replication in cells and especially re-P12.

**Figure 3.**
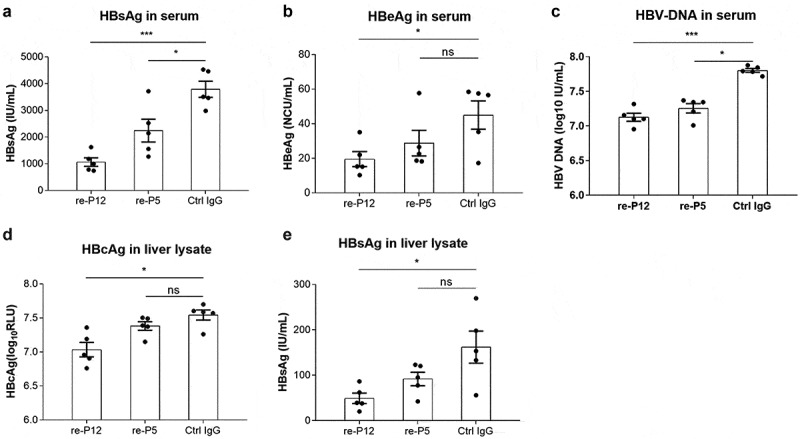
Evaluation of the HBV suppressive effects of recombinant mAbs in mice following co-injection of mouse recombinant antibody plasmids and HBV plasmids through the tail vein. The levels of HBsAg (**A**), HBeAg (**B**) and HBV DNA (**C**) in the serum were quantitatively measured by commercial assays on day 3 following hydrodynamic injections. The levels of HBcAg (**D**) and HBsAg (**E**) in liver lysates were quantitatively measured. The dosage of recombinant antibody plasmids and HBV plasmids was 10 μg per mouse. The data are expressed as the mean ± SD (n = 5 per group). P values were calculated using a two-sided unpaired t test, *Indicates P < 0.05, ***Indicates P < 0.001 and ns indicates P > 0.05

To further validate the therapeutic potential of recombinant anti-HBV pol antibodies, we evaluated the in vivo therapeutic effects of recombinant anti-HBV pol antibodies in a hydrodynamic injection (HDI)-based HBV mouse model. By hydrodynamic injection in the tail vein, 10 μg of antibody plasmid and 10 μg of the pAAV-HBV1.2 plasmid were injected into C57BL/6 mice. After 3 days of growth, the virology indices were assessed. re-P12 could reduce the levels of HBsAg, HBeAg, HBcAg and HBV DNA significantly both in the culture medium and in the cell lysates (Figure 3A–E). In contrast to these observations, re-P5 could only reduce the HBsAg and HBV DNA levels in the supernatants (Figure 3A, C).This result indicated that P5 and P12, notably P12 exhibited specific antiviral effects in vivo.

## Discussion

HBV pol is a viral nuclear protein that is difficult to detect [18,19]. The methods that can be used to detect HBV pol are limited and cannot meet the current research requirements. Therefore, more effective methods are required for HBV pol functional research. It has been reported that HBV pol is more easily detected when it is overexpressed in cells alone, whereas when HBV pol is expressed by the HBV genome, it becomes difficult to detect either by immunofluorescence assays or by WB analysis [20]. The reason for this finding may be that the amount of HBV pol protein expressed by the HBV genome is too small, whereas the sensitivity of the antibody is too low. Other studies have also shown that HBcAg inhibits immunofluorescence detection of HBV pol in cells and that this type of inhibition is independent of HBV capsidization [21,22]. Consistent with other studies, the antibodies produced in the current study were also unable to detect HBV pol, which was expressed by the HBV genome as demonstrated by immunofluorescence analysis. However, the antibody P20 was used to detect HBV pol expressed by the HBV genome, as demonstrated by WB analysis. This antibody exhibits considerable reactivity and can interact with four different HBV genotype pol enzymes (A, B, C, D). The results indicated that the structure of the polymerase enzymes was complex and that it may exhibit several linkages with other viral proteins, which can affect its binding to other antibodies. P20 is probably the most efficient antibody available for WB detection of HBV pol, which is expressed by the HBV genome. This antibody provided a new tool for further assessing the expression of HBV pol.

In recent studies, antibodies have been widely examined as biotherapeutic agents for a variety of diseases. In addition, their superior target specificity and low immunogenicity can enhance their efficacy as potential therapeutic drugs [23]. Although several antibodies exist in the market that target specific extracellular targets, their use in targeting intracellular targets is limited due to their inability to cross cell membranes. Recent advances in the development of methods for intracellular delivery of antibodies have resulted in the use of antibodies for targeting intracellular antigens [24–29]. As the field develops further, the boundaries between antibodies against intracellular and extracellular targets are not as clear as originally thought. Antibodies with novel mechanisms of action are challenging this belief and are redefining the selection of suitable targets for antibody therapy [30]. Antibodies that target intracellular antigens may open up new areas of therapeutic targets and may bring enormous clinical benefits. In the present study, transfection methods were used to deliver antibodies into cells, which was a relatively simple and easy to operate methodology. We investigated the antiviral effects of these antibodies in vitro and in vivo by transfection. These antibodies were expressed in Huh7 and liver cells of C57BL/6 mice. The results confirmed that P5 and P12 exhibited certain antiviral effects and notably re-P12. The data demonstrated that the antibodies screened in the current study could detect native HBV pol proteins and indicated that they could be used to identify novel targets for antibody-based drugs on HBV pol. However, additional studies should be conducted to safely and specifically introduce these antibodies in human liver cells.

In summary, the current study established an antibody denoted P20, which could be used to detect HBV pol expression by four HBV genomes via WB analysis. A new tool for studying HBV pol expression was identified. In addition, the data verified that the two antibodies denoted P5 and P12 could exert antiviral effects via intracellular expression notably P12, which may provide a promising approach for the treatment of chronic hepatitis B.

## Data Availability

The data that support the findings of this study are available from the corresponding author, Deying Tian, upon reasonable request.
